# Narrative review: updates and strategies for reducing door-to-balloon time in ST-elevation myocardial infarction care

**DOI:** 10.3389/fcvm.2025.1509365

**Published:** 2025-03-31

**Authors:** Xiaoru Zeng, Ling Chen, Ayush Chandra, Lin Zhao, Guanglong Ma, Francisco-Javier Roldan, Hongquan Wei, Wanling Pan, Wanquan Li

**Affiliations:** ^1^ Internal Medicine-Cardiovascular Department, Foshan Sanshui District People’s Hospital, Foshan, Guangdong, China; ^2^ Department of Clinical Medicine, Tianjin Medical University, Tianjin, China; ^3^ International Medical School of Tianjin Medical University, Tianjin, China; ^4^ Out-patient Department, National Institute of Cardiology, Mexico City, Mexico; ^5^ Department of 120 Emergency Command Center, Foshan Sanshui District People’s Hospital, Foshan, Guangdong, China; ^6^ Department of Nursing, Foshan Sanshui District People’s Hospital, Foshan, Guangdong, China

**Keywords:** STEMI, door-to-balloon time, pre-hospital strategies, in-hospital processes, technological innovations, quality improvement

## Abstract

This narrative review aims to evaluate strategies for reducing door-to-balloon (D2B) time in ST-elevation myocardial infarction (STEMI) patients, focusing on pre-hospital, in-hospital, and technological innovations, as well as addressing challenges to ensure sustainability. We reviewed recent literature from 2004 onward, examining various approaches to streamline STEMI care and improve D2B time. The review encompasses pre-hospital interventions such as advanced ECG monitoring and pre-hospital alerts, in-hospital strategies such as standardized protocols and streamlined processes, and technological innovations such as automated ECG interpretation, mobile applications, and telemedicine. Pre-hospital strategies have demonstrated significant benefits in reducing D2B times through early diagnosis and rapid communication. In-hospital approaches, including the use of clinical pathways and decision support systems, contribute to minimizing delays and enhancing coordination among healthcare providers. Technological innovations, such as automated ECG systems and telemedicine, facilitate quicker diagnosis and treatment initiation. However, challenges such as resource limitations, staff turnover, and variability in care processes persist. Addressing these challenges through continuous quality improvement, standardized care protocols, and data-driven analytics is crucial for sustaining improvements. Effective reduction in D2B time in STEMI care requires a multifaceted approach involving pre-hospital and in-hospital strategies, as well as leveraging technological advancements. Overcoming challenges and ensuring sustainability demands ongoing commitment to quality improvement, resource management, and standardized protocols. By integrating these strategies, healthcare systems can enhance the timeliness of STEMI care and improve patient outcomes.

## 1 Introduction

ST-elevation myocardial infarction (STEMI) is a severe manifestation of acute coronary syndrome characterized by complete occlusion of a coronary artery, leading to significant myocardial damage if untreated. Timely reperfusion therapy, primarily achieved through percutaneous coronary intervention (PCI), is essential. The success of PCI is heavily dependent on the rapid restoration of blood flow to the occluded artery, which is often measured by the door-to-balloon (D2B) time. D2B time is the duration between a patient's arrival at the hospital and the inflation of a balloon catheter within the blocked artery to restore blood flow. Reducing D2B time is critical, as every 30 min delay in treatment is associated with a 7.5% relative increase in one-year mortality for STEMI patients (1).

Over the years, various strategies have been developed and implemented to reduce D2B time. These strategies include pre-hospital interventions, in-hospital workflow optimizations, and system-level changes designed to expedite care. Pre-hospital strategies focus on early diagnosis and triage, often utilizing tools such as telemedicine and pre-hospital electrocardiograms (ECGs) to activate the catheterization laboratory before the patient arrives at the hospital (2). In-hospital strategies include streamlining emergency department processes, enhancing interdepartmental communication, and empowering multidisciplinary teams to respond rapidly to incoming STEMI cases (3). System-level interventions involve the establishment of regional STEMI networks, where hospitals work together to ensure the rapid transfer and treatment of STEMI patients (4).

Despite the proven efficacy of these strategies, there remains significant variability in D2B times across different institutions, regions, and countries. Understanding the factors that contribute to these disparities is essential for further improving patient outcomes. This literature review aims to critically examine the existing strategies for reducing D2B time in STEMI patients, highlighting the most effective approaches and identifying areas where further improvements can be made. By synthesizing findings from recent studies, this review provides insights into best practices and emerging trends in the management of STEMI, with the ultimate goal of reducing treatment delays and improving survival rates.

## 2 Clinical importance of reducing door-to-balloon time

Reducing D2B time in patients with STEMI is of paramount clinical importance because of its direct impact on patient outcomes, particularly mortality and morbidity. Timely reperfusion through PCI is critical for minimizing the extent of myocardial injury, preserving left ventricular function, and improving overall survival rates. Over the past decade, numerous studies have reinforced the association between shorter D2B times and improved clinical outcomes, underscoring the necessity of ongoing efforts to optimize these timelines (1, 5–8).

### 2.1 Impact on mortality and morbidity

One of the most compelling reasons for reducing D2B time is its significant influence on mortality. Studies consistently demonstrate that each minute of delay in PCI is associated with an increase in both short-term and long-term mortality. A meta-analysis revealed that patients with D2B times of less than 90 min had significantly lower in-hospital mortality rates than did those with longer D2B times (5). This effect is particularly pronounced in high-risk patients, such as those with anterior myocardial infarction or cardiogenic shock, where delays can exacerbate myocardial damage and increase the likelihood of fatal outcomes (9).

Furthermore, prolonged D2B times are associated not only with increased mortality but also with increased morbidity. Delays in reperfusion lead to larger infarct sizes, which in turn results in a reduced left ventricular ejection fraction, a predictor of future heart failure and arrhythmic events (10). As such, reducing D2B time is critical for preserving myocardial function and reducing the incidence of adverse cardiovascular events post-STEMI.

### 2.2 Improvements in functional recovery and quality of life

Reducing D2B time has also been shown to improve functional recovery and quality of life in STEMI survivors. Shorter reperfusion times contribute to better myocardial salvage, which is crucial for maintaining the structural and functional integrity of the heart muscle. This not only reduces the risk of heart failure but also leads to better functional outcomes, allowing patients to return to their daily activities with fewer limitations (6). Patients who received PCI within 90 min had improved clinical outcomes, including reduced mortality and better myocardial salvage, compared to those with longer D2B times (6).

### 2.3 Cost-effectiveness of reducing D2B time

In addition to direct clinical benefits, reducing D2B time is also cost-effective from a healthcare perspective. Shorter D2B times are associated with shorter hospital stays and lower rates of rehospitalization due to complications such as heart failure (11). This not only improves patient outcomes but also reduces the overall cost burden on healthcare systems. Additionally, hospitals that have successfully implemented strategies to reduce D2B time often see improved performance metrics, which can lead to higher reimbursement rates and better patient satisfaction scores. A protocol that reduced D2B time from 113.5 to 75.5 min resulted in a decrease in hospital length of stay from 5 to 3 days. Another study showed a reduction in length of stay from 4.4 to 3.6 days following a quality improvement initiative (7–12).

### 2.4 Current guidelines and benchmarking

Recognizing the clinical importance of reducing D2B time, professional guidelines have established strict benchmarks for its management. The American College of Cardiology (ACC) and the American Heart Association (AHA) recommend a D2B time of 90 min or less as the standard of care for patients with STEMI undergoing PCI (13). Meeting these guidelines is associated with improved patient outcomes and is a key quality indicator for hospitals providing acute coronary care.

Despite these guidelines, achieving optimal D2B times remains challenging, and there is ongoing variability in performance across institutions. This variability underscores the need for continuous quality improvement initiatives and the adoption of evidence-based strategies tailored to individual hospital settings.

## 3 Pre-hospital factors affecting the duration of STEMI

### 3.1 Age factors

Elderly patients with STEMI often experience longer treatment durations. This may be attributed to several factors. First, elderly patients frequently present with comorbid chronic conditions, which can complicated diagnosis and treatment. Second, they may exhibit atypical STEMI symptoms, including decreased pain sensitivity, potentially leading to delayed recognition of cardiac events. Age-related structural and physiological changes in the cardiovascular system, such as reduced vascular compliance and altered neuro-humoral regulation (14), further contribute to challenges in managing STEMI in this population. Myocardial ischemia in elderly patients is associated with more pronounced reperfusion disorders and increased risk of cardio-myocyte remodeling, predisposing them to STEMI (15). Finally, family members concerns about invasive procedures, sometimes coupled with a misunderstanding of the benefits of timely intervention, may contribute to treatment delays in elderly patients.

### 3.2 Gender factors

While the risk of STEMI increases with age in both men and women, STEMI is more prevalent in men (16). Although the mechanisms by which emotional stress and heavy physical activity might trigger STEMI are not fully understood, these factors can contribute to cardiac event in both the sexes. However, most women consider gynecological tumors as objects of concern, lack an understanding of cardiovascular-type diseases, and believe that they are high-incidence diseases in males; thus, they are mistaken for the typical symptoms of STEMI as other chronic diseases (17). At the same time, women usually have a high sense of family responsibility. They often ignore their own symptoms of discomfort as the center of life. There are also some concerns about the expenses of medical treatment and the methods of invasive treatment, resulting in delays in medical treatment time and first-aid operations.

### 3.3 BMI factors

BMI (Body-Mass Index) can be used as another key factor affecting the treatment time of STEMI patients. When the BMI is high, especially when the patient is obese, the possibility of myocardial infarction is significantly increased. Obesity has also been reported to be an important cause of myocardial infarction (3–5).

## 4 Pre-hospital strategies

Pre-hospital strategies play a critical role in reducing D2B time for patients with STEMI. By initiating diagnostic and therapeutic processes before the patient reaches the hospital, these strategies can significantly shorten the time to reperfusion, ultimately improving clinical outcomes. Advances in pre-hospital care, including the use of pre-hospital ECGs, telemedicine, and emergency medical services (EMS) activation protocols, have demonstrated considerable success in expediting STEMI treatment.

### 4.1 Pre-hospital electrocardiograms

The use of pre-hospital ECGs by EMS personnel is one of the most effective strategies for reducing D2B time. Pre-hospital ECGs allow for the early identification of STEMI, which can expedite the activation of the catheterization laboratory before the patient arrives at the hospital. This strategy has been shown to reduce both door-to-diagnosis and door-to-balloon times, leading to faster treatment and better patient outcomes.

A study revealed that pre-hospital ECGs were associated with a significant reduction in D2B times and in-hospital mortality among STEMI patients. Specifically, patients who had a pre-hospital ECG had median D2B times that were 15 min shorter than those who did not (18). This early identification allows for the activation of the catheterization team while the patient is en route, thereby minimizing delays once the patient reaches the hospital. Similarly, a systematic review supported these findings, showing that pre-hospital ECGs not only reduce D2B times but also improve overall survival rates in STEMI patients (2).

### 4.2 Telemedicine and pre-hospital notification

Telemedicine, including the transmission of pre-hospital ECGs to the receiving hospital, further enhances the effectiveness of early diagnosis and treatment initiation. By enabling real-time consultation between EMS personnel and hospital-based cardiologists, telemedicine facilitates immediate decision-making and ensures that the catheterization lab is prepared for the patient's arrival. A landmark study demonstrated that the transmission of pre-hospital ECGs via telemedicine reduced D2B times by up to 30 min. This approach allows the hospital to pre-activate the catheterization lab and mobilize the necessary staff and resources before the patient arrives, significantly reducing treatment delays (19). Moreover, the integration of telemedicine into pre-hospital care has been associated with a reduction in major adverse cardiovascular events, emphasizing its role in improving patient outcomes (20).

### 4.3 EMS activation protocols

Optimizing EMS protocols is another crucial strategy for reducing D2B time. Standardized protocols that prioritize rapid transport and minimize on-scene time can significantly shorten the interval between first medical contact and reperfusion therapy. Protocols often include criteria for bypassing non-PCI-capable hospitals and transporting STEMI patients directly to facilities equipped with PCI capabilities.

A study highlighted the impact of regionalized STEMI care networks, where EMS protocols are designed to ensure direct transport to PCI-capable centers. This study revealed that patients who were transported directly to a PCI-capable hospital had significantly shorter D2B times and lower mortality rates than those who were initially taken to a non-PCI hospital (21). The development of such regionalized systems, coupled with training for EMS personnel, ensures that patients receive the most appropriate and timely care.

Additionally, EMS systems that empower paramedics to initiate certain aspects of STEMI care, such as administering aspirin or other anti-platelet agents, can further reduce delays in treatment. According to one study, paramedic-initiated treatment protocols were associated with reduced D2B times and improved patient outcomes, highlighting the importance of early, protocol-driven interventions (22).

### 4.4 Public awareness and education

Public awareness campaigns and community education about recognizing the symptoms of STEMI and the importance of calling for emergency services promptly also play vital roles in reducing D2B time. The sooner patients activate EMS sooner pre-hospital strategies can be employed, ultimately reducing the time to treatment.

A study demonstrated that public education initiatives, coupled with pre-hospital ECG use, led to significant reductions in both symptom-to-first medical contact and D2B times. This highlights the interconnectedness of public awareness and pre-hospital strategies in optimizing STEMI care (23).

### 4.5 Challenges and future directions

While pre-hospital strategies have significantly improved D2B times, challenges remain in their implementation. Variability in EMS training, regional differences in healthcare infrastructure, and disparities in access to telemedicine can affect the consistency and effectiveness of these strategies. Future research should focus on addressing these challenges by standardizing pre-hospital care protocols across regions, expanding the use of telemedicine, and ensuring equitable access to advanced pre-hospital care for all populations.

## 5 In-hospital factors affecting the treatment time for STEMI

### 5.1 Type 2 diabetes factors

Patients with STEMI and type 2 diabetes, they are prone to a decrease in pain sensitivity due to autonomic nerve damage, which causes the typical symptoms of STEMI chest pain to be masked, resulting in a missed diagnosis and delays in treatment. Furthermore, patients with type 2 diabetes often exhibit more severe coronary artery disease, compromised cardiac function and more rapid progression of their condition. Compared with that of other people, the number of people with dyspnea as a symptom of STEMI in type 2 diabetes patients is significantly greater. Special attention should be given during clinical diagnosis and treatment.

### 5.2 Severity factors of hospital disease

The severity of the disease can directly affect the difficulty of treatment, thus interfering with the treatment time. If a patient has multiple diffuse lesions, the disease is more serious, which increases the difficulty of reperfusion in direct PCI treatment and increases the treatment time.

## 6 In-hospital strategies

In-hospital strategies are crucial for minimizing D2B time in patients with STEMI. These strategies involve optimizing processes within the emergency department (ED), streamlining interdepartmental communication, and ensuring rapid activation of the catheterization laboratory. Recent studies highlight the importance of a coordinated, multidisciplinary approach to reduce treatment delays and improve outcomes.

In collaboration with various hospital departments, such as emergency, cardiology, radiology, and nursing departments, the D2B time is significantly reduced, leading to quicker and more effective reperfusion therapy, which is crucial for STEMI patients. The study underscores the importance of teamwork in acute medical care to optimize results (24).

### 6.1 Streamlining emergency department processes

One of the most effective in-hospital strategies for reducing D2B time is the implementation of streamlined protocols within the ED. Early recognition of STEMI and prompt activation of the catheterization lab are essential steps. Protocols such as “STEMI alert” systems have been widely adopted to expedite these processes. When a STEMI is identified via an ECG, an automatic alert is sent to the catheterization team, reducing the time to lab activation.

A study demonstrated that implementing a STEMI alert protocol significantly reduced D2B times. The study revealed that hospitals with well-defined protocols achieved median D2B times of less than 60 min compared with hospitals without such protocols (25). The authors also emphasized the importance of regular staff training and simulations to ensure protocol adherence and to maintain a high level of preparedness.

In addition to alert systems, minimizing the time spent on nonessential tasks in the ED can also reduce D2B time. This includes bypassing time-consuming processes such as redundant paperwork or unnecessary imaging studies before the patient is transferred to the catheterization lab. Notably, hospitals that streamlined ED processes experienced significant reductions in both D2B time and mortality rates (26, 27).

### 6.2 Enhancing interdepartmental communication

Effective communication between the ED, the catheterization lab, and other relevant departments is critical for reducing D2B time. Multidisciplinary collaboration and the use of standardized communication tools can prevent delays that arise from miscommunication or unclear roles during the treatment of STEMI patients. The introduction of dedicated STEMI coordinators or liaisons has been shown to improve communication and coordination among the various teams involved in the care of STEMI patients. For example, hospitals with STEMI coordinators had significantly lower D2B times. These coordinators play a key role in ensuring that all team members are informed and ready to act as soon as the patient arrives (28).

Additionally, real-time communication technologies, such as secure messaging systems and dedicated STEMI platforms, allow for rapid dissemination of critical information. A study highlighted the effectiveness of secure messaging systems in reducing communication-related delays, leading to faster activation of the catheterization lab and shorter D2B times (29).

### 6.3 Rapid activation of the catheterization laboratory

Timely activation and preparation of the catheterization lab is a cornerstone of reducing D2B time. Strategies to ensure rapid lab activation include pre-hospital notification systems, parallel processing, and the use of dedicated, on-call catheterization teams.

One approach to rapid lab activation is the pre-hospital activation model, where the catheterization lab is notified and prepared as soon as a STEMI is identified in the pre-hospital setting. This strategy significantly reduces the time between patient arrival and the start of PCI. A study demonstrated that pre-hospital activation reduced D2B times by an average of 20 min, which was associated with improved patient outcomes. Another effective strategy is parallel processing, where multiple tasks are carried out simultaneously to reduce delays. For example, while the patient is being prepared in the ED, the catheterization team is concurrently preparing the laboratory (30). This approach contrasts with sequential processing, where each step is completed before the next step begins, often resulting in longer D2B times. Hospitals employing parallel processing had significantly shorter D2B times than those using traditional sequential workflows (2).

### 6.4 Empowering multidisciplinary teams

Empowering multidisciplinary teams, including emergency physicians, cardiologists, nurses, and technicians, to take initiative and make decisions is another important strategy. Protocols that allow ED physicians to activate the catheterization lab without waiting for cardiologist consultation can reduce unnecessary delays. Additionally, training all team members to respond swiftly and effectively to STEMI alerts ensures that each role is fulfilled without hesitation. One study emphasized the role of team-based care in reducing D2B time. The study revealed that hospitals where ED physicians were authorized to directly activate the catheterization lab had significantly shorter D2B times. The study also highlighted the importance of regular training sessions to ensure that all team members are familiar with the protocols and can act decisively during a STEMI event (22).

### 6.5 Initiatives for continuous quality improvement

Continuous quality improvement (CQI) initiatives are essential for sustaining reductions in D2B time. These initiatives involve regularly reviewing performance metrics, identifying bottlenecks in the treatment process, and implementing targeted interventions to address these issues. Hospitals that engage in CQI often see ongoing improvements in D2B times and patient outcomes. For example, a study discussed how hospitals participating in the American College of Cardiology's D2B Alliance saw progressive reductions in D2B times through continuous monitoring and iterative improvements in their processes. The study highlighted the importance of leadership support and a culture of accountability in driving successful CQI initiatives (28).

### 6.6 Challenges and future directions

Despite the success of in-hospital strategies, challenges such as resource limitations, staff turnover, and variability in protocol adherence can impact the effectiveness of these interventions. Future research should focus on developing standardized protocols that can be adapted across different hospital settings, as well as exploring the use of artificial intelligence and predictive analytics to further optimize in-hospital processes.

## 7 Technological innovations

Technological innovations have significantly contributed to reducing D2B time in the management of STEMI. These advancements facilitate faster diagnosis, streamline communication between healthcare providers, and optimize the coordination of care. Technologies such as automated ECG interpretation, mobile applications, telemedicine, and data-driven analytics are increasingly being integrated into workflows to increase the efficiency and effectiveness of STEMI treatment.

### 7.1 Automated ECG interpretation and transmission

Automated ECG interpretation and transmission systems have emerged as crucial tools in the early identification and management of STEMI. These systems are designed to automatically analyze ECG data, identify signs of STEMI, and promptly transmit the results to the receiving hospital or on-call cardiologist. This process ensures that the catheterization laboratory is prepped and that the appropriate clinical team is mobilized well before the patient arrives at the hospital. A study demonstrated that the use of automated ECG systems coupled with pre-hospital transmission significantly reduced D2B times. The study revealed that a patient who's ECGs were automatically transmitted and interpreted en route to the hospital had D2B times reduced by an average of 20 min compared with those without such technology (31). This reduction is crucial for improving outcomes, as each minute saved can reduce mortality and morbidity in STEMI patients.

Additionally, the integration of machine learning algorithms into ECG devices has further increased the accuracy and speed of STEMI diagnosis. For example, algorithms that continuously learn and adapt from previous data can improve the detection of subtle ECG changes indicative of myocardial infarction, leading to faster decision-making and lab activation (32).

### 7.2 Mobile applications and decision support systems

Mobile applications and decision support systems have revolutionized how healthcare providers manage STEMI cases, particularly in improving communication and coordination. These technologies allow real-time sharing of patient information, seamless communication between emergency medical services (EMS) and hospital teams, and quick access to clinical guidelines and protocols. The implementation of mobile applications specifically designed for STEMI management has been associated with substantial reductions in D2B time. A study revealed that the use of a mobile app that facilitated direct communication between the EMS, ED staff, and catheterization team reduced D2B times by up to 15 min (33). The app allowed EMS personnel to transmit pre-hospital ECGs, patient data, and estimated arrival times, ensuring that the hospital team was fully prepared by the time the patient arrived.

Moreover, decision support systems integrated into these apps provide evidence-based recommendations and reminders to clinicians, reducing variability in care and ensuring adherence to best practices. These systems can prompt clinicians to administer appropriate medications or activate the catheterization lab on the basis of real-time data, further reducing delays (34).

### 7.3 Telemedicine and remote monitoring

Telemedicine has emerged as a critical component in the effort to reduce D2B time by enabling remote diagnosis and management of STEMI. Through telemedicine platforms, EMS providers can transmit ECG data and other vital information directly to cardiologists or emergency physicians, who can then remotely confirm the diagnosis and initiate the necessary treatment protocols. A study highlighted the effectiveness of telemedicine in reducing D2B times. The study revealed that hospitals utilizing telemedicine for STEMI management achieved D2B times that were on average, 30 min shorter than those that did not (35). The ability to engage a cardiologist in real time, even before the patient arrives at the hospital, is a significant advantage in reducing delays and improving patient outcomes.

In addition to acute care, telemedicine facilitates continuous remote monitoring of patients with high cardiovascular risk, allowing for early detection of STEMI and rapid intervention. Remote monitoring devices can track vital signs; detect arrhythmias, and alert healthcare providers to potential issues, enabling preemptive action (36).

### 7.4 Data-driven analytics and predictive modeling

The use of data-driven analytics and predictive modeling in healthcare is transforming the management of STEMI by identifying patterns and predicting outcomes on the basis of vast amounts of data. These technologies can analyze historical D2B data, patient demographics, and clinical variables to identify factors that contribute to delays in care. By understanding these factors, hospitals can implement targeted interventions to address specific bottlenecks in the treatment process. Predictive modeling tools can also be used to forecast D2B times on the basis of real-time data, allowing hospitals to allocate resources more efficiently and anticipate challenges before they arise. For example, a study demonstrated that predictive analytics could be used to optimize the scheduling and availability of catheterization lab staff, leading to reductions in D2B times, especially during peak hours (37).

Furthermore, the integration of artificial intelligence (AI) with predictive analytics holds great promise for the future of STEMI management. AI can continuously learn from new data, improving the accuracy of predictions and enabling more personalized treatment approaches (29, 38). For example, AI algorithms can predict which patients are at greater risk of experiencing delays in care and prompt early interventions to prevent these delays.

### 7.5 Challenges and future directions

While technological innovations have greatly improved the ability to reduce D2B time, several challenges remain. The implementation of these technologies requires significant investment in infrastructure, training, and ongoing maintenance. Additionally, there is a need for standardized protocols to ensure the seamless integration of these technologies into existing workflows. Future research should focus on overcoming these challenges by developing cost-effective solutions that can be widely adopted across various healthcare settings. Moreover, as new technologies emerge, ongoing evaluation is necessary to assess their impact on patient outcomes and to refine their use in clinical practice.

## 8 Addressing challenges and ensuring sustainability

While significant progress has been made in reducing D2B time for STEMI patients, several challenges must be addressed to sustain these improvements over the long term. These challenges include resource limitations, staff turnover, variability in care processes, and the need for ongoing quality improvement initiatives. Addressing these challenges is crucial for ensuring that the gains made in reducing D2B time are not only maintained but also continuously improved upon.

### 8.1 Resource limitations

One of the primary challenges in reducing D2B time is the availability of resources, including both physical infrastructure and trained personnel. Hospitals, especially those in rural or underserved areas, may face difficulties in maintaining the necessary resources for rapid STEMI management. This includes ensuring that catheterization laboratories are available 24/7 and that skilled staff, such as interventional cardiologists and support personnel, are on hand to respond promptly to STEMI patients. Resource limitations can lead to delays in care, particularly during off-hours, when staffing may be reduced. A study highlighted that hospitals with limited resources often struggle to maintain short D2B times, especially during nights and weekends. To address these issues, some hospitals have adopted strategies such as cross-training staff to perform multiple roles, utilizing telemedicine to provide remote support, and forming regional STEMI networks where smaller hospitals can transfer patients to larger centers with more resources (39).

Additionally, innovative approaches such as mobile catheterization labs and partnerships with larger institutions can help mitigate resource constraints. For example, a study reported that regional collaboration and the use of mobile cath labs significantly reduced D2B times in resource-limited settings, leading to better patient outcomes (40).

### 8.2 Staff turnover and training

High staff turnover and the need for continuous training present ongoing challenges in sustaining reduced D2B times. Frequent changes in personnel can lead to variability in the execution of protocols and may result in delays. Moreover, the dynamic nature of STEMI care, which often involves the implementation of new technologies and protocols, necessitates ongoing education and training for all team members. Hospitals that prioritize continuous education and simulation training have been more successful in maintaining low D2B times. A study emphasized the importance of regular training sessions, which not only ensure that new staff are quickly brought up but also help reinforce best practices among existing team members (41). Simulation exercises, in particular, allow staff to practice responding to STEMI cases in a controlled environment, improving their readiness and coordination during actual events.

To address the challenge of staff turnover further, some hospitals have implemented mentoring programs where experienced clinicians guide new hires through the nuances of STEMI care. Such programs help maintain a high standard of care even as personnel changes occur (42).

### 8.3 Variability in care processes

Variability in care processes is another significant challenge that can undermine efforts to reduce D2B time. Differences in how STEMI protocols are implemented across departments or shifts can lead to inconsistencies in care delivery, resulting in delays. Standardizing care processes and ensuring adherence to protocols are essential for minimizing this variability. To address this issue, many hospitals have adopted clinical pathways and standardized order sets that outline the steps to be followed in the care of STEMI patients. These tools help ensure that all team members, regardless of their shift or department; follow the same evidence-based practices. A study demonstrated that hospitals with standardized STEMI protocols achieved more consistent D2B times and better patient outcomes than those without such protocols (3, 43).

Moreover, engaging all stakeholders in the development and review of these protocols can help identify potential sources of variability and address them proactively. Regular audits and feedback sessions can also be used to monitor adherence to protocols and make necessary adjustments to improve consistency (44).

### 8.4 Initiatives for ongoing quality improvement

Ensuring the sustainability of reduced D2B times requires a commitment to CQI. CQI initiatives involve the regular collection and analysis of data to identify areas for improvement and the implementation of targeted interventions to address these areas. Hospitals that have embraced CQI as part of their culture have been more successful in sustaining and further reducing D2B times. One study highlighted the importance of CQI in STEMI care. The study revealed that hospitals participating in CQI initiatives, such as the American College of Cardiology's D2B Alliance, not only achieved significant reductions in D2B times but also maintained these reductions over time (11). The key components of successful CQI initiatives include leadership commitment, multidisciplinary collaboration, and the use of performance metrics to guide improvements.

In addition to traditional CQI methods, the integration of data analytics and predictive modeling can further increase these efforts. By leveraging large datasets, hospitals can identify trends and patterns that may not be apparent through manual review. Predictive models can also help anticipate potential delays and guide real-time decision-making to prevent them (33).

### 8.5 Addressing disparities in care

Another important aspect of ensuring the sustainability of reduced D2B times is addressing disparities in care. Research has shown that certain patient populations, such as those from lower socioeconomic backgrounds or minority groups, may experience longer D2B times and worse outcomes (45). Addressing these disparities requires targeted interventions that consider the social determinants of health and aim to provide equitable care to all patients. Strategies to address disparities include culturally competent care, community outreach programs, and the use of patient navigators who can help guide underserved populations through the healthcare system. Additionally, ensuring that all patients have access to timely STEMI care, regardless of their geographic location or socioeconomic status, is crucial for improving overall outcomes (5).

### 8.6 Challenges and future directions

While much progress has been made, future efforts should focus on addressing the remaining challenges in sustaining low D2B times. This includes developing innovative solutions to overcome resource limitations, enhancing staff training programs, standardizing care processes across diverse healthcare settings, and continuing to engage in CQI initiatives. By doing so, healthcare systems can ensure that improvements in STEMI care are not only maintained but also built upon for future generations.

## 9 Conclusion

D2B time in STEMI patients is critical for improving outcomes and reducing mortality. This literature review highlights a range of strategies across various domains—pre-hospital, in-hospital, and technological innovations—and challenges—to achieve this goal. Pre-hospital strategies, such as early identification through advanced ECG monitoring and coordinated pre-hospital alerts, have proven effective in significantly reducing D2B times. Innovations in technology, including automated ECG interpretation, mobile applications, and telemedicine, further increase the speed and accuracy of STEMI diagnosis and treatment. These technologies facilitate real-time communication between EMS and hospital teams, ensuring rapid preparation and intervention. In-hospital strategies focus on streamlining emergency department processes, enhancing interdepartmental communication, and optimizing the activation of catheterization laboratories. Protocols such as STEMI alerts, parallel processing, and the use of decision support systems play a vital role in minimizing delays. The integration of these strategies helps maintain consistency in care and ensures timely response to STEMI patients. Despite progress, several challenges remain, including resource limitations, staff turnover, and variability in care processes. Addressing these challenges requires ongoing quality improvement initiatives, standardization of protocols, and robust training programs. Ensuring sustainability involves leveraging data-driven analytics to continuously refine practices and adopting innovative solutions to overcome resource constraints. In summary, while significant advancements have been made in reducing D2B time, the path forward requires a concerted effort to address existing challenges and sustain improvements. By continuously evolving strategies, incorporating new technologies, and fostering a culture of quality improvement, healthcare systems can further enhance the timeliness of STEMI care and improve patient outcomes.
